# Brilliant Blue Dyes in Daily Food: How Could Purinergic System Be Affected?

**DOI:** 10.1155/2016/7548498

**Published:** 2016-10-19

**Authors:** Leonardo Gomes Braga Ferreira, Robson Xavier Faria, Natiele Carla da Silva Ferreira, Rômulo José Soares-Bezerra

**Affiliations:** ^1^Laboratory of Inflammation, Oswaldo Cruz Foundation, Av. Brazil, 4365 Rio de Janeiro, RJ, Brazil; ^2^Laboratory of Toxoplasmosis, Oswaldo Cruz Foundation, Av. Brazil, 4365 Rio de Janeiro, RJ, Brazil; ^3^Laboratory of Cellular Communication, Oswaldo Cruz Foundation, Av. Brazil, 4365 Rio de Janeiro, RJ, Brazil

## Abstract

Dyes were first obtained from the extraction of plant sources in the Neolithic period to produce dyed clothes. At the beginning of the 19th century, synthetic dyes were produced to color clothes on a large scale. Other applications for synthetic dyes include the pharmaceutical and food industries, which are important interference factors in our lives and health. Herein, we analyzed the possible implications of some dyes that are already described as antagonists of purinergic receptors, including special Brilliant Blue G and its derivative FD&C Blue No. 1. Purinergic receptor family is widely expressed in the body and is critical to relate to much cellular homeostasis maintenance as well as inflammation and cell death. In this review, we discuss previous studies and show purinergic signaling as an important issue to be aware of in food additives development and their correlations with the physiological functions.

## 1. Introduction

The purinergic receptor superfamily has ionotropic and metabotropic receptors. These receptors are widely expressed in the body and shows distinct pharmacological properties and activation pathways [1, 2]. The G protein-coupled P2Y receptor subtypes are activated by adenosine triphosphate (ATP), uridine triphosphate (UTP), and metabolites, such as adenosine diphosphate (ADP) and uridine diphosphate (UDP). There are eight mammalian subtypes: P2Y1R, P2Y2R, P2Y4R, P2Y6R, P2Y11R, P2Y12R, P2Y13R, and P2Y14R. Depending on the type of G protein coupled to the P2Y receptors, its activation triggers different signaling cascades. In general, these events lead to phospholipase C recruitment, inositol 3-phosphate formation, and intracellular Ca^2+^ release from intracellular stores, as well as modulating adenylyl cyclase-related signaling [3, 4]. On the other hand, the mammalian ATP-gated ion channels, namely, P2X, are composed of the following 7 subtypes: P2X1R, P2X2R, P2X3R, P2X4R, P2X5R, P2X6R, and P2X7R [4]. Following their activation, the P2X receptors lead to rapid mobilization of monovalent and divalent cations, such as K^+^, Na^+^, and Ca^2+^, which depolarize plasma membrane and trigger several intracellular events. Nevertheless, because of the lack of selective agonists, synthetic ATP analogues were designed, such as 3′-O-(4-benzoyl)benzoyl-ATP and adenosine 5′-[*γ*-thio]triphosphate, for pharmacological experiments. Conversely, P2YR subtypes have distinct agonist preferences. There are receptors preferentially activated by ATP, such as P2Y11R; those preferentially activated by ADP, such as P2Y1R, P2Y12R, P2Y13R; those preferentially activated by UDP, such as P2Y6R; and those preferentially activated by UTP, such as P2Y2R and P2Y4R. Yet, antagonists development has been highly prolific. There are many categories of blockers that have been properly described and used against both P2Y and P2X receptors [1, 5–8]. It is noteworthy that many diseases display purinergic signaling involvement, in which several researchers have focused on new pharmacological strategies targeting P2 receptors [5]. Indeed, Gum and colleagues have stated in 2012 some challenges in development of orally purinergic receptors specific drugs and achieving suitable bioavailability [9].

**Figure 1:**
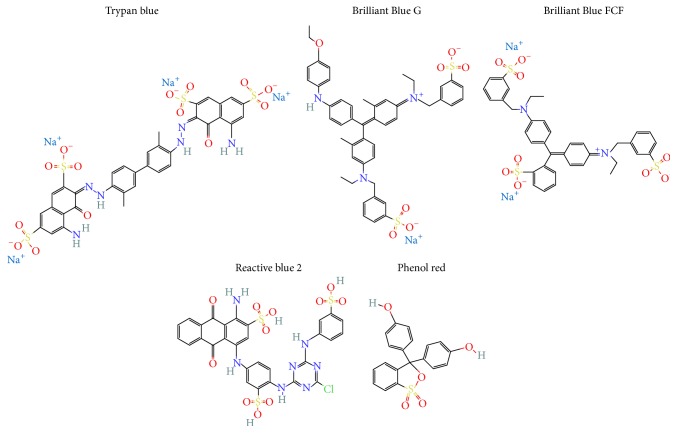
Structure of the dyes, which are P2 receptors antagonists.

Curiously, many available P2 receptor antagonists have biological and industrial uses. Brilliant Blue G (BBG) [10], reactive Blue 2 (RB-2) [11], phenolphthalein (Phenol red) [12], and trypan blue [13], shown in Figure 1, are widely used. Among these first compounds mentioned as purinergic inhibitors, they lack pharmacological selectivity, which, in turn, might inhibit one or more subtypes of P2 receptors and unexpectedly modulate some organ/tissue functions (via P2 receptor inhibition). Nevertheless, only BBG (P2X7 antagonist) was assessed* in vivo* (and in humans) and it has utility in the clothing and food industries. Therefore, as P2X7 has critical roles under both physiological and pathological circumstances as inflammation, infection, and tissue injury [5], this review comes up with warnings about such compound ordinary uses and purinergic signaling.

## 2. Industrial Uses of Brilliant Blue G and Its Derivative

The Brilliant Blue dye family has several members in which BBG and FD&C Blue No. 1 have wide uses in health sciences and industrial issues. The synthetic dye Brilliant Blue G, also known as Coomassie Brilliant Blue, was first synthesized from coal tar dye. It has a reasonable stability when exposed to light, heat, and acidic conditions, whereas it has low oxidative stability. On the other hand, FD&C Blue No. 1 (also known as the Brilliant Blue FCF or E133 in the European numbering system) (Figure 1) is one of the most common dyes used in food and cosmetic preparations and medicines. FD&C Blue No. 1 was approved in various countries to be used as a food additive in dairy products, candies, cereals, cheese, toppings, jellies, liquors, and soft drinks. This dye is also used in cosmetics such as shampoos, nail polishes, lip gloss, and lip sticks and in the textile sector [14]. The uses of this dye are justified due to its high cost-benefits as blue is not a color currently found in secretions in the body [15]. It is noteworthy that FD&C Blue No. 1 is also found in green shaded food and drinks as a mixture with yellow dyes. It has a low gastrointestinal absorption, and the amount absorbed is highly excreted by biliary vesicles and urinary routes [16]. However, FD&C Blue No. 1 utilization was not allowed in some countries, such as Germany, Austria, France, Belgium, Norway, Sweden, and Switzerland before European Union foundation. In other countries, such as the United States, its use is unconditional; in Canada, use is limited to 100 ppm; in England, it can be used in some food; in the European Union, it is allowed for any use [17], and in Brazil, consumption is allowed up to 100 g [18]. Prado and Godoy analyzed the concentrations of different dyes by HPLC, including FD&C Blue No. 1 in different types of food in Brazil. Chocolates and candies contained an amount of the dyes within the limits, whereas in gumballs, the FD&C Blue No. 1 content was above the authorized concentration [18]. This fact may be more complicated when one considers alimentary imported products, whose specifications do not conform to the current Brazilian legislation [18]. FD&C Blue No. 1 has been related to skin irritations and bronchial constriction, especially when combined with other dyes [19]. In this sense, FD&C Blue No. 1 may exhibit another impairment to health, such as high genotoxic properties due to DNA base intercalation.

## 3. Brilliant Blue Dyes* In Vitro* Assays

FD&C Blue No. 1 has been linked to intracellular enzyme modulation, such as protein tyrosine phosphatases (PTPases). PTPases, along with protein tyrosine kinases, regulate the cellular phosphorylation levels [20]. Shrestha and colleagues studied the action of some food color additives on the activity of these phosphatases* in vitro*. FD&C Blue No. 1 inhibited PTP1B function (IC_50_ = 91 *μ*M) while the enzymes TC-PTP and YPTP1 were less sensitive than PTP1B with IC_50_ values greater than 120 *μ*M [21]. Furthermore, FD&C Blue No. 1 has been suggested to inhibit mitochondrial respiration* in vitro*, which was shown to aggravate sepsis by enteral tube feedings [22].

At the bench, Brilliant Blue dyes, more specifically BBG, have been applied in electrophoresis studies, especially for protein visualization. The advantage of these triphenylmethane dyes is that they give strong colored bands when they combine with a cellulose acetate membrane. BBG has been extensively used in several biochemical analyses and protein quantification in protein-dye binding assays [23–26]. Moreover, alternative colorimetric enzyme activity assays were proposed based on BBG properties [27–30]. Similarly, Brilliant Blue R is usually applied in protein analyzers, mainly in electrophoresis and in microscopy [31–33]. In light microscopy procedures, BBG was used to visualize intracellular organelles in hepatocytes and fibroblast cell cultures [34, 35]. BBG is an ingredient of the Bradford assay, which biochemically measures intracellular protein [36]. Additionally, this dye was also used to purify virus-like particles subjected to sucrose density ultracentrifugation. This technique allows for the identification of rubella virus-like particles [37].

Nevertheless, as mentioned above, BBG is a known noncompetitive antagonist for P2X7R [10, 38]. There are many studies showing that BBG has a higher selectivity and potency to rat P2X7R with an IC_50_ of 12 nM [10], but BBG also inhibited human P2X7R at a nanomolar range (IC_50_ = 265 nM) [10]. BBG was also shown to be efficient in blocking P2X7R-induced cytotoxicity in retinal cells [39], microglial cells [40], and astrocyte cells [41]. However, there are divergences concerning the applicability of BBG in human cells. Eschke and colleagues demonstrated a reduction in activity in human macrophage cells following BBG exposure [42].

Alternatively, FD&C Blue No. 1 and its analogue FD&C Green No. 3 have been shown to selectively inhibit pannexin-1 in oocytes-transfected system. Interestingly, both inhibitors showed the same IC_50_ in an oocytes heterologous system (IC_50_ of 0.27 *μ*M) and were ineffective as a P2X7R inhibitor [43]. The pannexin-1 is widely expressed in the organism and it has been connected to the P2X7R-induced pore-forming phenomenon, whereas this point remains controversial [44, 45]. Nevertheless, this relationship should not be ignored, once this appears to be upon cell type dependence, as in astrocyte. Indeed, Garre et al. showed that BBG inhibited ethidium uptake in astrocyte similarly to _10_Panx1, a mimetic inhibitory peptide of pannexin-1 [46]. However, supposing that pannexin-1 is part of the P2X7-pore phenomenon, which part of this complex is inhibited should be carefully assessed and one cannot affirm that BBG is specifically blocking pannexin-1. Furthermore, there are several indications that pannexin-1 is associated with ATP release and the inflammasome assembling, indicating that it could be related to inflammatory diseases, such as colitis and Crohn's disease [43, 47–52]. In 2011, Jo and Bean observed that BBG was a sodium channel blocker in mouse N1E-115 neuroblastoma cells, whereas FD&C Blue No. 1 had little effect on sodium channel [53].  Table 1 shows major Brilliant Blues dye effects* in vitro* assays.

## 4. Brilliant Blue Dyes* In Vivo* Assays

BBG should also be carefully studied in this context once it is currently used clinically in ophthalmic procedures [54, 55]. Surely, it is reasonable to consider that inflamed (mostly chronic) tissues would show P2X7R hyperactivity (directly or indirectly by pannexin-1) and that the ingestion of food containing blue dye could relieves or diminishes deleterious processes by P2X7R inhibition [43, 51, 56]. In addition, Mennel and colleagues studied some vital dyes to visualize anatomical structures during vitreoretinal surgery in 2008. BBG had no effects on the morphology and functionality of the optical tissues. Additionally, the effect of BBG in the retinal pigment epithelium (RPE) was analyzed* in vitro*. The transepithelial resistance (TER) was used to measure the barrier integrity after 3 days of BBG treatment. In the fluid-filled eye model, BBG at 0.25 mg/mL did not affect the outer blood retinal barrier. The concentration of 2.4 mg/mL in the fluid-filled eye and air-filled eye models showed a decrease after 1.5 h, which was no longer observed after 24 h. In addition, there were no structural alterations of the RPE cells after BBG treatment. Clinically, BBG did not stain epiretinal membranes, and it represents an appropriate candidate for the future, as BBG has a high affinity for the internal limiting membrane [57]. In vitreoretinal surgical procedures, Höing and collaborators used BBG as a macular surgery stain. Patients received it during vitrectomy for macular holes or epiretinal membranes. The authors analyzed several parameters, such as best corrected visual acuity and intraocular pressure, perimeter, fundus photography, and optical coherence tomography. At the end, they concluded that BBG sufficiently and selectively stained the internal limiting membrane (ILM). Additionally, there was no retinal toxicity or side effects associated with the BBG, and the safety of long-term ingestion should be evaluated in a larger patient series and in a longer follow-up [58]. Based on these preliminary studies and molecular structural similarities among BBG and other Brilliant Blue food dyes, Franke and collaborators investigated purinergic precipitates in microglial activation in the rat nucleus accumbens (NAc)* in vivo* based on extracellular ATP release after brain injury. Using confocal image analysis, they observed increases of P2X1, P2X2, and P2X4 and P2Y1, P2Y2, P2Y4, P2Y6, and P2Y12 subtypes in the region of the lesion, mainly related to P2X7R immunoreactivity, which colocalized with active caspase 3, but not with the antiapoptotic marker pAkt. P2R agonists augmented the immunoreactivity of P2R and P2X7R. BzATP administered intra-accumbally increased the caspase-3 activity. In contrast, PPADS and BBG diminished injury and immunoreactivity [59]. According to Mennel et al.'s paper evaluating FD&C Blue No. 1* in vivo*, Peng et al. studied the effect of BBG on traumatic spinal cord injury. The authors gave 10 or 50 mg/kg BBG per day, immediately after injury and for three consecutive days. This treatment did not promote any effects on behavior, weight, survival, or other physiological parameters, including body temperature, blood pH, blood gases, or blood pressure. The evaluation of the blood-brain barrier permeability of BBG in the setting of a medullary lesion was quantified, and following a 10 mg/kg-dose, they measured 9.94 *μ*M BBG within contused spinal cord tissue. At a dose of 50 mg/kg of BBG, a concentration of 43.59 *μ*M was achieved in the lesion 3 days later [60].

As described above, to study neuroinflammation, they produced a lesion in the spinal cord, which led to an instantaneous and irreversible loss of tissue at the contusion point and consequent enlargement of tissue injury over time. Although secondary injury should be potentially avoidable, the patients with acute spinal cord injury (SCI) do not have adequate medication options. After the first lesion, the traumatized tissue releases ATP leads to P2X7R activation. The authors applied BBG systemically in a weight-drop model of thoracic SCI in rats to evaluate the neuroprotective reactions. BBG administered for 15 days after the SCI suppressed spinal cord anatomic damage and improved motor restoration without apparent toxicity. The local BBG application inhibited local activation of astrocytes and microglia and neutrophil infiltration [60].

Díaz-Hernández and collaborators studied the reduction of ATP production in* Drosophila* models of Huntington's disease (HD), which is associated with synaptic disturbances and the elevation of neuronal apoptosis. In this initial paper, they demonstrated the influence of the P2X7R on apoptosis triggered by activation of its receptor* in vitro*. The administration of the BBG in HD mice,* in vivo*, impaired the symptomatology, such as body weight loss, motor-coordination deficits, and neuronal apoptosis [61].

In 2010, Andó et al. compared the analgesic activity of antagonists acting at P2X1R, P2X7R, and P2Y12R and agonists acting at P2Y1R, P2Y2R, P2Y4R, and P2Y6R in neuropathies associated with neurogenic and inflammatory pain. BBG and other drugs were evaluated by mechanical allodynia in the Seltzer model of neuropathic pain, by acute thermal nociception, and by the inflammatory pain and edema induced by complete Freund's adjuvant (CFA). BBG only presented a moderate effect on inflammatory pain [62].

Also in 2010, Okamoto and collaborators analyzed the toxicity of BBG using* Drosophila melanogaster*. They evaluated the long-term toxic effects of continuous and a single exposure of* Drosophila melanogaster* to BBG added to the culture medium. BBG concentrations did not affect the survival of the insect model. Because BBG concentrations higher than 15 mM cannot be evaluated (BBG was insoluble), LC_50_ was estimated to be 38 mM. Additionally, BBG was not neurotoxic [63].

P2X7R is abundantly expressed on microglia; this cell type is capable of modulating long-term potentiation (LTP) of spinal pain. Possibly, this process occurs through regulating the communication between microglia and neurons. Additionally, microglial cells are the main components that activate the spinal LTP, which are the C-fiber induced field potentials generated by tetanic stimulation of the sciatic nerve (TSS). In this context, Chu and colleagues investigated the function of the P2X7R in the LTP evoked by TSS in rats.* In vitro*, BBG and oxidized ATP diminished the TSS stimulation of spinal LTP in spinal cord slices. In turn,* in vivo* BBG administration inhibited the induction of spinal LTP and diminished mechanical allodynia. The intrathecal application of BBG blocked the upregulation of microglial P2X7R. However, IL-1*β* expression was inhibited in the model of LTP evoked by TSS pretreated with BBG. This result was confirmed by a reduction in the expression of this cytokine after pretreatment with an IL-1 receptor antagonist (IL-1ra) [64].

Isolated retinal ganglion cells (RGCs), glia, and other cell types surrounding the ganglion cells may evoke stimulatory or inhibitory effects through P2X7R activation. Hu and collaborators investigated the participation of the P2X7R in retinal ganglion cell death. BzATP was applied intravitreally in the superior nasal region of rats in the presence or absence of BBG and MRS 2540. Both antagonists suppressed ganglion cell death [65].

In 2011, another paper demonstrated the involvement of the P2X7R in the pain mechanism. They studied the mustard oil-induced glutamatergic-dependent central sensitization, a mechanism present in medullary dorsal horn (MDH) nociceptive neurons of the tooth pulp. In this model, the central sensitization occurs due to an increase of the neuronal excitability of nociceptive pathways with subsequent peripheral tissue injury and inflammation. The expression of P2X7R was assessed in the presynaptic terminal and glial cells, and in the latter, its association in chronic inflammatory and neuropathic pain was evaluated. In this paper, the authors described P2X7R participation in the acute inflammatory pain model evaluated in anesthetized rats [66]. For the study of the nociceptive neurons in the MDH, they measured unitary records of mechanoreceptive fields, the mechanical activation threshold, and responses to noxious stimuli. Mustard oil- (MO-) induced MDH central sensitization was reduced by continuous intrathecal superfusion of both BBG and oxidized ATP. Interestingly, the microglial blocker minocycline also diminished the MO-induced MDH central sensitization [66]. According to the authors, these results confirm that dorsal horn P2X7R is expressed on microglia and that P2X7R may be connected to central sensitization in an acute inflammatory pain model [66].

In 2012, Arbeloa et al. evaluated P2X7R participation in neuronal excitotoxicity. In primary neuronal cultures and in brain slices, electrophysiological and Ca^2+^ assays recorded BzATP inhibition by BBG administration. In addition, the neuronal death promoted by oxygen-glucose deprivation was impaired by BBG treatment. The formation of a middle cerebral artery occlusion in rats was used as a model of transient focal cerebral ischemia. The area of brain damaged after the ischemia was diminished in the animals pretreated with BBG, compared to treatment with vehicle alone [67].

Also in 2012, Chu et al. studied P2X7R action in neuroinflammation observed in cerebral ischemia/reperfusion (I/R) injury. In this context, they used the rat model of transient global cerebral I/R injury. Transient global cerebral I/R was induced using the four-vessel occlusion (4-VO) method after 20 minutes of infusion with BBG, oxidized ATP (oxATP), or A-438079. The high dosage of BBG (10 *μ*g) and A-438079 (3 *μ*g) and low dosage of oxATP protected against transient global cerebral I/R injury in a dosage-dependent manner, augmented survival rates, retarded I/R-induced learning and memory deficits, and suppressed I/R-induced neuronal death, DNA cleavage, glial activation, and inflammatory cytokine overexpression in the hippocampus [68].

In 2012, Kimbler and colleagues assessed the effects of BBG, orally administered, in traumatic brain injury (TBI), which is associated with posterior cerebral edema. Neurosurgical procedures to ameliorate the edema-induced augmented intracranial pressure are questionable, and there are no efficient drugs to treat this symptom. Using P2X7R knockout mice (P2X7^−/−^ mice), the expressions of IL-1*β* and cerebral edema were impaired. In another strategy to inhibit P2X7R, BBG (25 mg/kg) was administered via the drinking water for one week before the TBI induction or via an intravenous bolus four hours after the TBI. Both strategies diminished the TBI effects. The final BBG concentration in the plasma was quantified after its intravenous administration. They measured a BBG value of 383 *μ*M and 1.73 mM following the application of 50 mg/kg and 100 mg/kg, respectively. These results implicate P2X7R as a therapeutic target to avoid the secondary effects of TBI [69].

As mentioned above, P2X7R activation leads to diverse intracellular signaling, such as CD62L expression pathways in different cell types [70]. In the paper published by Cascabulho and collaborators, they studied Duchenne muscular dystrophy (DMD) using a mouse model (*mdx/mdx* mouse). This model presents most aspects of the disease, such as a low quantity of T cells in damaged muscles, the degeneration of skeletal and cardiac muscles, and chronic inflammation. The authors investigated the migration of T cells to the heart and disturbances in adhesion molecules in the* mdx/mdx* mouse model. They demonstrated a reduction in the positive CD62L expression in blood leukocytes, including T cells, of six-week-old* mdx/mdx* mice. A downregulation and reduction of approximately 40% of T cells expressing CD62L in 12-week-old mice were observed. This diminished CD62L quantity was associated with the suppression of the ability of blood T cells to adhere to cardiac vessels* in vitro* and reach cardiac tissue* in vivo*. BBG treatment recovered the CD62L in blood lymphocytes, and they maintained their ability to migrate to the heart [71]. Another scientific group in 2012 studied the role of the P2X7R in DMD using the* mdx* mouse model. Dystrophies muscles had upregulated P2X7R mRNA and protein expression* in vitro*. They linked the protein quantity to the change in the responsiveness of intracellular Ca^2+^ and extracellular signal-regulated kinase (ERK) phosphorylation. Dystrophies mice exhibited alterations in P2X7R in isolated primary muscle cells and dystrophic muscles* in vivo*. Treatment with BBG impaired the number of degeneration-regeneration cycles in mdx skeletal muscles* in vivo*. Both papers suggested that the disturbance in P2X7R expression and function occurs in dystrophic* mdx* muscle. In addition, treatment with P2X7R antagonists, such as BBG, may reverse this effect [72].

There are reports of neuronal injuries in a large number of neurodegenerative disturbances. These damages, in some cases, are related to P2X7R activation. In prion-related diseases, there are lethal neurodegenerative disturbances that do not have effective therapies. Based on this information, Iwamaru and collaborates stated that the blockade of the P2X7R could impair prion replication conjointly as a therapeutic tool for prion infection. The administration of BBG reduced the agglomeration of pathogenic prion protein (PrPres)* in vitro*. In addition, the brain of infected mice had a diminished number of PrPres after the administration of BBG* in vivo* [73]. It is noteworthy that recently BBG was shown to prevent neuronal loss in mouse models and modulates amyloid-*β* aggregation and cytotoxicity in cell-based assays, suggesting a new therapy for AD [74, 75].

In 2013, Kakurai and colleagues investigated the action of the P2X7R in cultured retinal ganglion cells after optic nerve crush (ONC) injury. P2X7R agonists reduced the viability of RGCs* in vitro*, which was reversed with P2X7R antagonist pretreatment. Using rats with ONC injury, BBG suppressed impairments in the vitreous body by approximately 61% of baseline 7 days after the lesion was generated [76].

A relevant function associated with P2X7R activity is the activation of the inflammasome, such as NLRP3. In this context, lupus nephritis (LN) is composed of inflammatory and autoimmune events. Interestingly, NLRP3 may modulate both characteristics. Additionally, the binding between P2X7R and the NLRP3 inflammasome pathway was investigated in the pathogenesis of LN. The authors used the mouse lupus model, MLR/*lpr*, and the parameters analyzed were anti-dsDNA antibody production, survival, activation of the NLRP3/ASC/caspase-1 inflammasome, and the Th17/Treg ratio on renal lesions. The MLR/*lpr* mice presented a substantial upregulation of the P2X7/NLRP3 inflammasome compared to nontreated mice. Following 8 weeks of BBG treatment, the treated mice exhibited P2X7R inhibition and diminished NLRP3/ASC/caspase-1 assembly and IL-1*β* release. Consequently, there was suppression of the nephritis, circulating anti-dsDNA antibody levels, and an augmented survival of the mice. Moreover, the IL-1*β*, IL-17, and Th17/Treg ratio levels in the serum were impaired with BBG administration. Similar results to those with BBG were observed after silencing P2X7R using interference RNA* in vivo* [77].

Menzies and collaborators studied chronic renal dysfunctions. This pathology is globally prevalent in approximately 10% of the population. In this work, they reported that the Fischer (F344) rat had a lower glomerular filtration rate (GFR) than the Lewis rat and is more susceptible to renal injury induced by hypertension. They performed kidney activity microarray assays to find candidate genes for impaired blood pressure control using the endeavor enrichment tool. The* P2rx7* and* P2rx4* genes showed seven- and threefold increased expression, respectively, in F344 rats. These purinergic receptors were visualized in the endothelium of the preglomerular vasculature and in the renal tubule, and this expression was similar in both rat species. The BBG treatment of Lewis rats did not affect the blood pressure but elevated renal vascular resistance (possibly due to inhibition of some basal vasodilatory tone). Both parameters were diminished in F344 rats (possibly due to an increase in the vasoconstrictor tone). In addition, BBG suppressed the diuresis pressure threshold in F344 rats [78].

Among the diverse functions attributed to P2X7R, the receptor may induce motor neuron death in brain alterations. Based on amyotrophic lateral sclerosis (ALS), which is the progressive degeneration of motor neurons that is currently without treatment, the relationship between P2X7R and ALS was investigated by Cervetto et al. in 2013. The transgenic mice have mutations causing superexpression of the superoxide dismutase 1 gene, which was utilized to study the influence of the gender, disease course, motor performance, weight loss, and life span. The administration of BBG inhibited the progression of ALS [79].

As an attempt to reduce the toxic action of the trypan blue (TB) dye, a combination containing TB, Brilliant Blue G (BBG), and polyethylene glycol had been used in ARPE retinal pigment epithelial cells. In this paper, the authors inquired the toxic effect promoted by this association. TB and BBG were exposed alone or in combination with ARPE cells. TB in concentrations above 0.075% and BBG in concentrations above 0.1% were toxic to the cells after 30 min incubation. In the case of BBG, in concentrations of 0.1% and higher, it showed a protective effect on cells when incubated by 5 min. Concentration of 0.025% BBG was able to preserve against TB-induced damage at 5 min and 30 min incubation [80].

Also in 2013, Henrich, as principal investigator of University Hospital, Basel, Switzerland, patented the clinical trial entitled “Intraoperative Utility of Brilliant Blue G (BBG) and Indocyanine Green (Icg) Assisted Chromovitrectomy”. Although Icg is the dye more indicated to color the ILM in a surgical extraction, in comparison to BBG, it is not approved to intravitreal use, because there are description of ocular toxicity caused by its utilization. In this clinical trial, the medicines intend to find hypothetical alterations in Icg and BBG dyes to obtain improved intraoperative dye utility associated with a safety profile (ClinicalTrials. NCT01485575) [81, 82].

In 2014, based on limited information about the relationship between dyes and the causes of retinal damage after ERM and ILM peeling, Giansanti and colleagues evaluated the toxicities of blue dyes on retinal pigment epithelium and retinal ganglion cells with light exposure (with and without halogen and xenon light exposure) [83]. Indeed, the researchers treated the human retinal pigment epithelium line ARPE-19 and the rat retinal ganglion cells RGC5 with 0.5% vital dyes for 5 min and exposed them to light. Cell viability was estimated by proliferation assay using WST-1 reagent for 12, 24, or 120 h after the light exposition. Time-lapse video microscopy recorded the morphological aspects of the apoptosis for 72 h. They did not observe toxicity to both cell lines, when they were exposed to light. ARPE-19 cells present moderate toxicity to BBG after xenon illumination, in contrast; RGC5 cell line did not exhibit toxicity to BBG.

Balaiya and collaborates studied the light-induced decomposition of vital dyes, in the case of the BBG, that occurring during the chromovitrectomy [84]. Phototoxic effects of the BBG have not yet been described, but the phototoxicity depends on the light source, the intensity of illumination, the distance of the light source from the surface of the retina, and the duration of exposure. Then, they investigated the BBG toxicity in human retinal pigment epithelium (HRPE) cells stimulated to metal halide surgical endoilluminator (SE) in different distances of illumination. BBG was used in the concentrations of 0.25 and 0.5 mg/mL on ARPE-19 cell line and illuminated with SE for 1, 5, and 15 min. The surgical distance of illumination was hypothesized varying distances (1 and 2.5 cm) of the illumination used. Cell viability and the distance of illumination of 1 cm during the times of 1 (about 90%), 5 (about 60%), and 15 min (about 35%) of exposure was similar to BBG treatment with doses of 0.25 mg/mL and 0.5 mg/mL. In contrast, the distance of 2.5 cm exhibited augment in the viability with 0.25 mg/mL BBG 1 min (98.85%–3.3%), 5 min (95.31%–7.12%), and 15 min (62.07%–3.0%). In consequence, they concluded that BBG in the concentration of 0.25 mg/mL during vitreoretinal surgery is not toxic to HRPE under focal illumination (1 cm) or diffuse illumination (2.5 cm).

As a potential food dye, FD&C Blue No. 1* in vivo *assays were carried out according to the Food and Drug Administration (FDA) oral absorption and oral-intake limits, which in healthy animals was 12 mg/kg/day. However, in critical situations, such as during sepsis, there is a higher gastrointestinal permeability due to enterocyte death and a loss of barrier function at intercellular gaps. In this context, systemic absorption of FD&C Blue No. 1 from enteral feedings after tracheostomy for obstructive apnea and chronic renal failure have been described [22] and, in some cases, the systemic absorption of FD&C Blue No. 1 leads to death [85]. However, possibly because of its low oral absorption, only a few studies have documented toxicity related to FD&C Blue No. 1 in different tissues, as there are no evident alterations (even in carcinogenicity studies) in the animal models used [86, 87]. In addition, Abd El-Wahab and Moram studied some of the most used colorants and flavors and their toxic effects on essential organs. Increasing in FD&C Blue No. 1 intake altered some parameters, whereas it did not increase mass weight. Additionally, the ingestion of this dye was associated with an increase in important hepatic health-indicative enzymes, such as alanine transaminase, aspartate transaminase, alkaline phosphatase, and the amount of bilirubin [88].  Table 2 shows the main studies of the Brilliant Blue dye effects* in vivo* assays.

## 5. Dye Concentrations Used in the Food Industry and the Concentration to Inhibit P2 Receptor Function in Humans

Among the dyes with the capacity to inhibit the P2 receptors listed above, only BBG (or its analogue) is used in the food industry. Therefore, we will focus on this dye to investigate the possible correlations between the concentrations used in food and in scientific experiments.

In beverages, such as Gatorade® Lemon-Lime, the quantity of FD&C Blue No. 1 added in a bottle of 946 mL is 0.1 mg/L. In other types of Gatorade, this concentration is higher: Gatorade Glacier Freeze (1 mg/L), Gatorade Tropical Blend (2 mg/L), and Gatorade Blueberry Pomegranate (10 mg/L).

The Food and Drug Administration (FDA) investigates new food coloring (dye) when it is developed to analyze toxicology and safety studies, estimates human dietary intake, and then publishes literature for this drug and identifies an acceptable daily intake (ADI) level. The value calculated for FD&C Blue No. 1 according to body weight for an adult is 12.5 mg/kg/day. The EU Scientific Committee for Food (SCF) revised the ADI to 10 mg/kg/day in 1984, based on new long-term studies. In theory, an adult weighing 60 kg would tolerate a maximum amount of 720 mg of FD&C Blue No. 1. More recently, the Panel on Food Additives and Nutrient Sources determined that the no observed adverse effect level (NOAEL) of 631 mg/kg/day of the chronic toxicity study in rats could be used to designate another ADI for FD&C Blue No. 1. Applying an uncertainty factor of 100, the Panel supports a new ADI for FD&C Blue No. 1 of 6 mg/kg/day [EFSA Panel on Food Additives and Nutrient Sources added to Food (ANS), 2010]. However, practically, it is difficult to estimate the quantity of dyes consumed without information about the quantity of the dye used in common foods. Additionally, new studies about the impact of a prolonged intake of this dye and cancer and neurological disturbances in humans are necessary.

A number of papers have used BBG to inhibit P2X7R* in vivo*. BBG concentrations administered to the animals of the studies varied, in general, from 1 mg/kg to 100 mg/kg. Although a large number of scientific publications used this dye* in vivo*, among those found for us, only three quantify the final concentration of BBG in the body. In 2009, Peng and colleagues quantified BBG concentrations in the medullary tissue as 9.94 *μ*M after giving a dose of 10 mg/kg and 43.59 *μ*M after a dose of 50 mg/kg [60]. In the same year, Díaz-Hernández and coworkers also quantified BBG in the plasma after 45 mg/kg administration in one- and four-month-old mice. In the former, BBG was measured at 7.08 *μ*M in the plasma and 152.6 nM in the brain; in the 4-month-old mice, the plasma concentration was 0.04 *μ*M and 226.12 nM in the brain [61]. Kimbler and collaborates quantified the concentration in the plasma as 383 *μ*M and 1.73 mM of BBG after the administration of 50 mg/kg and 100 mg/kg, respectively [69].

Taking into consideration the results of the Kimbler and Díaz-Hernández groups, the final BBG concentration obtained is sufficient to functionally block P2X7R* in vivo* [61, 69]. Based on IC_50_ values,* in vitro* concentrations can inhibit rat P2X7R (10 nM), human P2X7R (270 *μ*M), and mouse P2X7R (170 nM). At a micromolar range, this antagonist may reach other P2X receptors, such as rat P2X2R with an IC_50_ of 1.4 *μ*M, rat P2X4R with IC_50_ of 5 *μ*M, human P2X4R with an IC_50_ of 3.2 *μ*M, and other channels, such as voltage-gated sodium channels with an IC_50_ of 2 *μ*M [43]. In this study, FD&C Blue No. 1 inhibited voltage-gated sodium channel, producing a modest effect compared to BBG [43]. More convincing results were published in cells transfected with pannexin-1, where this dye inhibited the pannexin-1 opening with an IC_50_ value of 0.27 *μ*M [43].

Diverse beverages and foods contain the maximum permitted level of FD&C Blue No. 1 ranging from 100 mg/L to 200 mg/L and 50 to 500 mg/kg, respectively (EFSA Panel on Food Additives and Nutrient Sources added to Food (ANS), 2010). However, studies on the FD&C Blue No. 1 concentration used in drinks and food have indicated a typical use concentration in the industry ranging from 0.1 to 60 mg/L. Moreover, researchers have also reported extreme use levels ranging from 0.9 to 200 mg/L and typical use levels ranging from 0.1 to 300 mg/L for beverages reported and reported typical use levels ranging from 0.2 to 500 mg/L for foods (DyeDiet, 2011; EFSA Panel on Food Additives and Nutrient Sources added to Food (ANS), 2010).

Using the example of an adult male weighing 60 kg and an adult female of 55 kg and the male having 70 mL/kg and the female 65 mL/kg of blood, we estimate a total blood volume of 4.2 L for the male and 3.9 L for the female. Using the reported typical use levels of beverages cited above as a reference, we could predict the quantity in milligrams of the dye for the lower limit as shown in Table 3. In a male weighing 60 kg, the calculation of the mass is 0.007 mg/kg and for a female of 55 kg the calculation is 0.007 mg/kg. In micrograms, this relation remains 7 *μ*g/kg for both genders. The upper limit is shown in Table 4. In a male weighing 60 kg, the calculation of the mass is 4.2 mg/kg and for a female of 55 kg the calculation is 4.25 mg/kg. With regard to the reported typical use levels of foods, the quantity in milligrams of the dye for the lower limit is the same as Calculus 1 (Table 2). In addition, the upper limit was calculated below. In a male weighing 60 kg, the calculation of the mass is 21 mg/kg and for a female of 55 kg the calculation is 21.27 mg/kg.

Evidently, the final intake of blue dyes in the alimentary is variable, depending on the portion of blue dye in each food and quantity of the food absorbed. Our intention is to warn consumers about the possibility of a functional inhibition of the P2 receptors, including P2X7R in particular, caused by food intake. We highlight that FD&C Blue No. 1 concentrations used in the alimentary described above may be superior to IC_50_ values that potently inhibit P2X7R (and other P2X), considering pannexin-1-dependent ATP releasing (IC_50_ = 0.27 *μ*M) and the voltage dependent sodium channels. There are diverse pathological situations favoring an augment in the gut and skin absorption and clinical procedures as enteral feeding. In these cases, the quantity of FD&C Blue No. 1 in the tissues increases in comparison to healthy patients. Nevertheless, it is noteworthy that scientific researches of dyes in food should not be limited to this area for cumulative absorption studies. These studies should not ignore the great amount of daily life products and have to account other pathways. In 2013, Lucová et al. [89] showed important assays of cumulative absorption through intact skin, shaven skin, and lingual mucosa. They showed that the skin integrity is relevant to systemic dye absorption, which significantly increases after shaving and epilation procedures. Hemorrhagic shock, nonsteroidal anti-inflammatory drug use, renal failure, inflammatory bowel disease, and cystic fibrosis, among others, are some diseases related to increase in the gut permeability [15]. Once P2X7R, in general, is upregulated under pathological conditions, including the cases cited above, possibly, BBG could achieve plasmatic concentration sufficient to inhibit P2X7R. Additionally, the intravenous administration of 50 mg/kg BBG in a mice model of traumatic brain injury promoted a plasmatic value of 383 *μ*M after 4 hours and the application of 100 mg/kg BBG produced a plasmatic value of 1.73 mM also after 4 hours [60]. This could be unfavorable to proinflammatory functions, while, in contrast, inhibition of the P2X7R* in vivo* would be central to the prevention of spine injury or Crohn's disease.

## 6. Conclusions

In this work, we discuss the effect of the two Brilliant Blue dyes related to purinergic signaling (BBG and FD&C Blue No. 1) that were widely used in many manufactured products until recently. Our purpose was to elucidate the link between the industry concentration usage to produce dyed food or medicines and its dosage in the human system. These studies have become necessary to elucidate the possible therapeutic or deleterious effects obtained through the ingestion of manufactured products, which could modulate some diseases related to P2R function. Surely, this work does not focus on the abolishment of food dyes but is to shed light on the putative relationship within food dyes and purinergic signaling. A few works have shown that restriction of synthetic food color additives has ameliorated some attention deficit or hyperactivity disorder symptoms [90–92]. Whereas there is no work linking purinergic signaling and deficit or hyperactivity disorder symptoms, it will not be a surprise if soon they appear. Indeed, there are many purinergic receptors in glial cells, which have been studied in these disorders [93–95]. Therefore, scientific researches should assess purinergic signaling and manufactured products (as food dyes) relationship and should not be omitted in several inflammatory and infectious diseases.

## Figures and Tables

**Table 1:** The main studies of the Brilliant Blue dyes effects *in vitro* and *in vivo* assays.

Brilliant Blue FCF				Ref.

*In vitro* assays	Phosphatases activities modulation	PTP1B function	IC_50_ = 91 *µ*M	[21]
		YPTP1 function	IC_50_ > 120 *µ*M	
	Selective pannexin-1 activity inhibition		IC_50_ = 0.27 *µ*M	[43]
	Mitochondrial respiration inhibition			[22]

*In vivo* assays	Food and Drug Administration (FDA) oral absorption limits		12 mg/kg/day	[22]
	Higher gastrointestinal permeability in sepsis		Systemic absorption enteral feedings after tracheostomy for obstructive apnea	[22]
			Systemic absorption chronic renal failure	[22]
			It leads to death	[85]
	No evident alterations (even in carcinogenicity studies) in the animal models used			[86, 87]
	Increase in important hepatic health-indicative enzymes		Alanine transaminase, aspartate transaminase, alkaline phosphatase, and bilirubin	[88]

Brilliant Blue R				

*In vitro* assays	Biochemical analyses and protein quantitation in protein-dye binding assays			[23–26]
	Electrophoresis studies microscopy			[31–33]
	Polyacrylamide gels in electrophoresis			[30]

Brilliant Blue G				

*In vitro* assays	Biochemical analyses and protein quantitation			[23–26]
	The administration of BBG reduced the agglomeration of pathogenic prion protein (PrPres) *in vitro*			[73]
	Electrophoresis studies			[31–33]
	Microscopy studies			[34, 35]
	Polyacrylamide gels in electrophoresis			[30]
	Bradford assay			[36]
	Virus-like particles purification			[37]
	P2X7R noncompetitive antagonist	IC_50_ = 12 nM (rat)		[10]
		IC_50_ = 265 nM (human)		[10]
		P2X7-induced calcium influx	pIC_50_ < 4 (mouse) pIC_50_ = 5.09 (rat) pIC_50_ < 4 (human)	[96]
		P2X7-associated pore formation (Yopro-1 uptake)	pIC_50_ = 6.71 (BALB/c mouse) pIC_50_ = 6.34 (C57BL/6 mouse) pIC_50_ = 6.24 (rat) pIC_50_ = 5.71 (human)	[96]
	Pannexin-1 antagonist	Oocytes-expressing Pannexin-1-induced ionic currents	IC_50_ ~ 3 *µ*M	[97]

*In vivo* assays	Ophthalmic procedures			[54, 55]
	Visualization of anatomical structures during vitreoretinal surgery			[57]
	Vitreoretinal surgical procedures			[58]
	Deleterious consequences of ATP release following brain injury	Microglial activation in the rat nucleus accumbens (NAc)		[59]
		Inhibition of ATP-induced caspase-3 activity		
	Traumatic spinal cord injury	Local astrocytes and microglia activity inhibition		[60]
		Inhibition of neutrophil infiltration		
	Huntington's disease (HD)	BBG impaired the symptomatology (body weight loss, motor-coordination deficits, and neuronal apoptosis)		[61]
	Seltzer model of neuropathic pain	Acute thermal nociception		[62]
	Freund's adjuvant (CFA) pain model	Moderate effect in inflammatory pain and edema		
	BBG toxicity assays in *Drosophila melanogaster*	No impairment on survival of the insect model (LC_50_ = 38 mM). No neurotoxic effects		[63]
	Suppression of P2X7R-induced ganglion cell death			[64]
	Protective neuronal effects upon oxygen-glucose deprivation	The brain damaged area following ischemia was diminished in the animals pretreated with BBG, compared to treatment with vehicle alone		[67]
	Cerebral ischemia/reperfusion (I/R) injury	BBG (10 *μ*g) protected against transient global cerebral I/R injury, augmented survival rates, retarded I/R-induced learning and memory deficits, and suppressed I/R-induced neuronal death, DNA cleavage, glial activation and inflammatory cytokine overexpression in the hippocampus		[68]
	Traumatic brain injury (TBI) induced by posterior cerebral edema	BBG (25 mg/kg) orally administered before the TBI induction diminished the TBI effects		[69]
	Duchenne muscular dystrophy (*mdx/mdx* mouse)	BBG treatment recovered the CD62L in blood lymphocytes and it maintained its ability to migrate to the heart		[71]
		Treatment with BBG impaired the number of degeneration-regeneration cycles in mdx skeletal muscles *in vivo*		[72]
	Agglomeration of pathogenic prion protein (PrPres)	Diminished number of PrPres in infected mouse brain after the administration of BBG		[73]
	Chronic renal dysfunctions renal injury induced by hypertension	BBG suppressed the diuresis pressure threshold in F344 rats		[78]
	Amyotrophic lateral sclerosis (ALS)	The administration of BBG inhibited the progression of ALS		[79]

**Table 2:** Calculus 1.

Male	Female
0.1 mg Brilliant Blue FCF, 1 L	0.1 mg Brilliant Blue FCF, 1 L
*X* mg Brilliant Blue FCF, 4.2 L	*Z* mg Brilliant Blue FCF, 3.9 L
*X* = 0.42 mg Brilliant Blue FCF	*Z* = 0.39 mg Brilliant Blue FCF

**Table 3:** Calculus 2.

Male	Female
60 mg Brilliant Blue FCF, 1 L	60 mg Brilliant Blue FCF, 1 L
*X* mg Brilliant Blue FCF, 4.2 L	*Z* mg Brilliant Blue FCF, 3.9 L
*X* = 252 mg Brilliant Blue FCF	*Z* = 234 mg Brilliant Blue FCF

**Table 4:** Calculus 3.

Male	Female
300 mg Brilliant Blue FCF, 1 L	300 mg Brilliant Blue FCF, 1 L
*X* mg Brilliant Blue FCF, 4.2 L	*Z* mg Brilliant Blue FCF, 3.9 L
*X* = 1260 mg Brilliant Blue FCF	*Z* = 1170 mg Brilliant Blue FCF
